# First Report on Occurrence and Characterization of Microplastics in Feces of *Larus armenicus* (Buturlin, 1934) in the Lake Van Basin (Eastern Anatolia, Türkiye)

**DOI:** 10.3390/toxics14030202

**Published:** 2026-02-27

**Authors:** Erkan Azizoğlu

**Affiliations:** 1 Department of Plant and Animal Production, Çölemerik Vocational School, Hakkari University, 30100 Hakkari, Türkiye; erkanazizoglu@hakkari.edu.tr; Tel.: +90-0507-749-31-92; 2 Center for Biodiversity Application and Research, Hakkari University, 30100 Hakkari, Türkiye

**Keywords:** microplastics, bird feces, ecotoxicology, FTIR, endorheic lake, *Larus armenicus*

## Abstract

Microplastics (MPs) are widespread worldwide and have become a significant environmental problem due to their durability and the large quantities that enter ecosystems. As the global spread of microplastic pollution continues, the Armenian gull (*Larus armenicus*) in the Lake Van Basin has emerged as an important bioindicator. This study highlights the widespread impact of human-generated waste on natural habitats by detecting the presence of microplastics in gull feces using a non-invasive, polymer-supported method. **Methods:** The study was conducted between 10 May 2024 and 26 April 2025. A total of 480 fecal samples were analyzed from four stations with different characteristics and exposed to various anthropogenic effects. Instead of individual-level statistical inference, we performed temporal comparisons descriptively at the composite level. **Results:** We categorized suspected MPs by type, shape, size, and color, using FTIR to confirm the polymer identity of a representative subset (>300 µm; ~20%) and SEM–EDX to examine particle surfaces. A total of 8197 MP particles were observed in the feces collected from the stations. The most frequently observed MP type, size, shape and color were fiber (32.6%), 100–300 µm (30.8%), spherical (29.2%) and brown (18.4%), respectively. The chemical structures of all examined MPs were polyethylene (PE) (42.6%), polystyrene (PS) (28.38%) and polyethylene terephthalate (PET) (8.5%). SEM-EDX confirmed that the microplastics are polymers by showing their degraded surface and carbon/oxygen ratio. **Conclusions:** Identifying polymer species in ingested plastics is valuable for future studies, as the results can be used to assess the relationship between microplastics.

## 1. Introduction

Plastic generally refers to a wide range of synthetic polymer materials that can be easily shaped and molded using heat, and because of properties such as easy shaping, insulation, light weight, and low production costs, these materials have been widely employed in daily life since the 1950s. Plastic has become a game-changing substitute for materials used in industry and society, profoundly altering the way healthcare is delivered as well as how different products are packaged, stored, and transported [1,2].

Plastics are widely used worldwide and have become a significant environmental problem due to their durability and the enormous amounts that enter ecosystems [3]. Moreover, the accumulation of polymers in the environment is not only a result of high production rates but also significantly driven by inadequate waste management practices and insufficient recycling infrastructures [4]. By 2050, it is anticipated that 33 billion tons of plastic will be produced worldwide [5]. It is estimated that about 1.15 to 2.41 million tons of plastic waste enters the ocean from rivers each year, carrying large amounts of inland waste [6,7,8,9]. Beyond riverine inputs, UNEP estimates that approximately 19–23 million tonnes of plastic waste leak into aquatic ecosystems annually, contaminating rivers, lakes, and oceans [10,11]. Therefore, plastic pollution is recognized as a serious anthropogenic problem in coastal and marine ecosystems worldwide [12]. Rather than undergoing biological decay, synthetic plastics are subject to continuous physical fragmentation, which yields persistent microplastic particles (MP < 5 mm). These derivatives resist natural degradation and result in an enduring environmental presence [13,14,15,16]. Microplastics are commonly defined as plastic particles < 5 mm spanning micrometer- to millimeter-scale sizes [17]. They originate as “primary” particles manufactured at small sizes (e.g., industrial pellets and microbeads) or as “secondary” particles formed by degradation and weathering of larger plastic products [18]. In many environmental matrices, fibers released from synthetic textiles and ropes frequently dominate counts, reflecting diffuse sources, such as domestic wastewater and atmospheric fallout [19,20]. Due to their small size and high density, plastic waste easily spreads into aquatic ecosystems through various human-induced processes, such as solid-waste facilities, wastewater treatment plants, industrial discharges, agricultural practices, direct release of plastic waste into the environment, the decomposition of large plastic pieces over time, and atmospheric transport [21,22,23].

MPs entering aquatic ecosystems can be ingested and absorbed by many organisms through their diet [24,25]. Organisms associated with aquatic environments are more exposed than other organisms. The ingestion or absorption of microplastics by these organisms poses a potential threat to aquatic ecosystems. Exposure can occur via direct ingestion, incidental intake with prey, and trophic transfer [26,27]. Plastic ingestion may cause physical harm (e.g., blockage and perforation) and may also facilitate exposure to additives or sorbed contaminants [28,29].

Wetlands are particularly vulnerable because they receive inputs from agriculture, settlements, runoff, and waste disposal and can function as sinks where MPs accumulate [24,25,30]. Evidence from intertidal ecosystems highlights a multi-compartmental contamination of sediments and biota, reinforcing the paradigm that wetland-mediated food webs act as a primary conduit for microplastic accumulation in avian species [30,31,32].

Many studies have attempted to identify microplastic contamination by utilizing gizzards and other digestive tract sections of bird carcasses [33,34,35,36,37,38]. Although microplastic occurrences are well-established across a myriad of avian biological matrices, the paradigm is shifting toward fecal-based monitoring as a non-invasive, ethically superior, and logistically versatile alternative to traditional sampling protocols [39]. Because destructive sampling of gastrointestinal tracts is ethically and logistically constrained for many species, non-invasive approaches such as fecal analysis are increasingly used to assess microplastic ingestion and egestion [40,41,42]. While MP particles in bird feather and pellet samples have not yet passed into the digestive system [43], MP particles in fecal samples are excreted already digested. MP particles in feces, in particular, directly affect birds because they are already digested [44]. Thus, the number of MP particles in feces appears to be directly proportional to the number of MP particles in the digestive tract [40]. Fecal-based monitoring can provide an ethical and scalable tool for comparing sites and seasons, especially when supported by robust QA/QC and polymer verification.

MP assessments based on fecal analysis have been effectively utilized across a broad range of taxa and habitats, encompassing urban waterbirds, shorebirds, seagull and avian communities within protected areas [40,41,42]. In particular, seabirds function as exemplary sentinel organisms for marine plastic pollution; their diverse foraging niches, heterogeneous feeding guilds, and broad trophic distribution allow for a comprehensive assessment of plastic infiltration across multiple marine strata [45,46]. Sea-, shore- and waterbirds are widely used as bioindicators because they integrate contamination across habitats, trophic levels, and large spatial scales [45,46]. Analysis of bird feces from a large protected wetland revealed that MP abundance fluctuates across ecological groups, underscoring the role of habitat use and diet in determining exposure risks.

As one of the avian groups most severely threatened by anthropogenic pressures [47], seabirds have become a focal point of conservation efforts alongside growing scientific awareness of marine microplastic pollution [48]. Already utilized as effective bioindicators for macroplastic pollution [49], these species hold similar potential for monitoring microplastics within both the environment and their ecologically and economically significant prey [50]. However, while the literature on macroplastic ingestion is extensive [51,52,53], microplastic interactions remain insufficiently documented across many species and ecosystems.

Gulls (family Laridae) frequently exploit anthropogenic food sources and can therefore be exposed to plastics, yet feces-based evidence remains scarce for several species and regions. The Armenian gull breeds across Eastern Türkiye, Armenia, Southern Georgia, and Northwestern Iran and stands as a dominant species in Türkiye’s Eastern Anatolia region. Its primary breeding stronghold is the Lake Van Basin, which encompasses numerous wetlands providing vital habitats for the species. Despite the availability of these critical areas, the breeding population in Türkiye has undergone a severe decline in recent years [54,55,56,57]. As a closed system, the Lake Van Basin acts as a significant sink for anthropogenic pollutants; recent studies have increasingly documented MP contamination across its surface waters and diverse aquatic habitats [58,59]. It is an endorheic alkaline lake system subject to tourism, urbanization, and local waste inputs. The lack of an external outlet suggests that these synthetic particles are sequestered within the basin, potentially intensifying the exposure risk for local biota.

As a top predator primarily breeding in high-altitude lacustrine ecosystems, such as Lake Van, *Larus armenicus* acts as a significant bioindicator for environmental health, reflecting the cumulative anthropogenic pressures on these semi-isolated basins. Its opportunistic feeding behavior—incorporating both aquatic organisms and anthropogenic waste—subjects the species to high rates of multi-pathway MP ingestion, a phenomenon increasingly documented in Laridae families globally [40]. Physiologically, the species’ high metabolic rate and site fidelity during breeding seasons increase the potential for localized bioaccumulation and systemic MP toxicity [60]. Consequently, investigating MP occurrence in *Larus armenicus* is essential not only for assessing the specific health of this regional population but also as a necessary proxy for understanding the trophic transfer of plastics within sensitive aquatic-terrestrial interfaces [61].

Despite being a species of conservation priority, the Armenian gull remains understudied regarding its interaction with MPs. Although the existing literature has documented plastic consumption in various Laridae species worldwide [40,52,53,62,63] the Armenian gull has been entirely overlooked. Consequently, no data currently exists on microplastic uptake in this species, even though significant populations aggregate annually in the Lake Van Basin for breeding. Therefore, investigating the frequency of microplastic ingestion by the Armenian gull is crucial for assessing the pollution risks faced by this species. Such an assessment serves as a fundamental starting point for evaluating feeding dynamics related to plastic uptake and underscores the potential of the Armenian gull as a bioindicator for this group of contaminants.

Accordingly, this study aimed to (i) quantify MP occurrence in *Larus armenicus* feces across four stations representing contrasting land use and anthropogenic pressure, (ii) describe particle size, shape and color distributions, and (iii) characterize polymer types and surface features using FTIR and SEM–EDX. We hypothesized that stations with higher human activity and waste inputs would exhibit higher MP loads in feces.

## 2. Materials and Methods

### 2.1. Study Area

**Figure 1:**
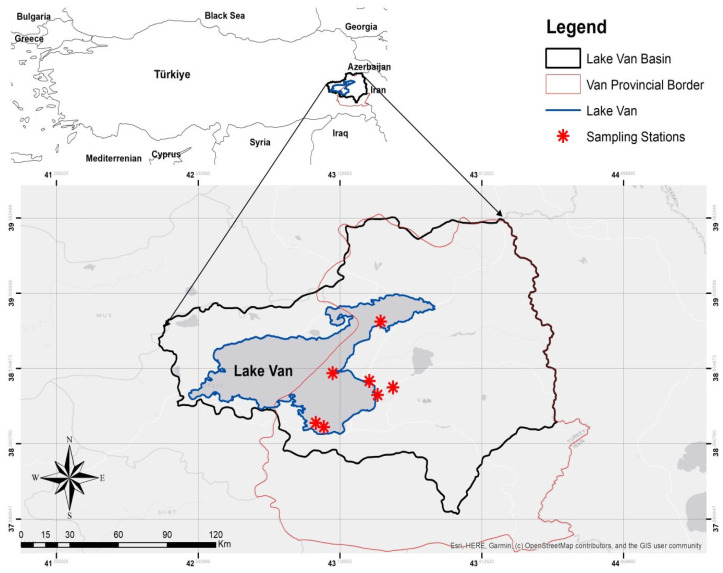
Location of the study area and sampling stations.

The research area selected was the province of Van, a large city covering an area of 19,069 km^2^ located in the Lake Van Basin, one of the important basins in Turkey’s Eastern Anatolia Region (Figure 1). The city where the study was conducted was chosen due to its location, its geography, the importance of its wetlands for birds and the fact that it hosts the largest breeding grounds of the Armenian gull. In addition, the indented structure of the shores of Lake Van, the fact that it hosts many large and small islands, islets and rocks, and special conditions such as the mixing of characteristically different waters are features that can increase the diversity and abundance of microplastics (MPs) in this region. Field sampling was carried out on 10–12 May 2024, 11–15 January 2025 and 25–26 April 2025 in Lake Sıhke (Bostaniçi) in the Lake Van Basin, the Lake Van islands (Akdamar, Çarpanak and Adır), the surroundings of Van Castle and the pond in the Campus (see Figure 1). Sample collection sites included Van Castle, which has recreational areas and agricultural fields shared by the people of Van; Sıhke Lake near the solid-waste facility; a campus area frequently used by people; and the islands of Lake Van, which are used for tourism and are breeding grounds for the species. The sampling areas facilitated the detection of the species’ feces because they were areas where the Armenian gull bred and roosted in large numbers.

### 2.2. Feces Sample Collection

In this study, a total of 723 Armenian gull feces were collected from four stations with varying land cover and human-induced impacts. The stations where feces were collected were observed in advance, and their geographic coordinates were recorded. In particular, to avoid sample duplication in areas with dense Armenian gull populations and where communal feeding and breeding grounds are located, fecal samples were collected simultaneously. Additionally, collecting fecal samples from a single location where numerous bird species are present was avoided [39,64,65,66]. After a 30 to 45 min waiting period, they went to the area where the flock had left the resting place. To prevent sediment contamination, only intact fecal deposits clearly separated from the substrate were selected for sampling. Where samples were found on soft ground, only the topmost, fresh portion of the deposit was collected, strictly avoiding the fecal–substrate interface. Furthermore, fecal samples were collected as sterilely as possible from rocky and stony areas, regardless of gender, age, size, or other characteristics. Immediately after collection, sample containers were sealed with aluminum-foil-covered lids to eliminate direct contact with plastic components and ensure sample integrity, and stored in ice-filled cloth bags [67]. After the sampling work was completed, the samples stored in ice bags were brought to the Hakkâri University Biodiversity Application and Research Laboratory and stored in deep freezers at −20 °C until analysis.

### 2.3. Sample Processing and MP Analysis

Fecal samples, which were stored at −20 °C in deep freezers, were thawed under laboratory conditions. To enhance the reliability of statistical comparisons and ensure equal sample sizes across groups, 120 samples were selected from each region using the simple random sampling method. This study set, comprising a total of 480 samples, was partitioned into 4 homogeneous subgroups per region, each containing an equal number of specimens. Consequently, the entire study data was organized into 16 homogeneous groups for subsequent analysis. After thawing, the samples were quantitatively transferred into sterile 800 mL beakers by rinsing with Milli-Q water to ensure no residue remained. The homogenized samples were then placed in glass beakers and dried in a 40 °C oven for 48 h to remove moisture. A 10% potassium hydroxide (KOH) solution was added to the moisture-free dry fecal samples to digest them at 50 °C on a hot plate for 48 h (Bessa et al., 2019; Bourdages et al., 2021 [68,69]). The floating materials were separated and filtered through a Whatman GF/A filter paper (47 mm diameter, 1.6 µm pore size) using a vacuum pump (Millipore; Burlington, MA, USA). A 10% hydrogen peroxide (H_2_O_2_) solution was added to the fecal residue in the glass beaker and kept at room temperature for 24 h to ensure complete organic digestion. After organic digestion, the sample was again filtered under vacuum through Whatman GF/A filter paper (Cytiva, Maidstone, UK) (47 mm diameter, 1.6 µm pore size) using the same procedures. Filter papers holding the filtered MPs were stored in 60 mm glass Petri dishes and left to dry at room temperature. The processed filter papers were then examined under a stereo microscope (Labomed Vision 2000, Labomed, Inc, Los Angeles, CA, USA) to identify potential MP candidates. The MP particles were counted under a microscope and photographed using ScopeImage software (Version 9.0, Windows 10). To define MPs more accurately, the guidelines of [70] Hidalgo-Ruz et al. (2012) were followed. A color scheme was used to define the colors of the MPs [51] (Provencher et al. 2019). The length and width measurements of the MPs were documented using ImageJ software (version 1.54r; NIH, Bethesda, MD, USA). The identified MPs were then categorized according to their color, shape (piece or fiber), and size (length and width). After observation, diagnosis, and photographing, MP particles larger than 300 µm were collected from filter papers using a fine needle under a microscope and placed in a sterile 1.5 mL glass vial for further analysis. To minimize contamination, all glass/metal equipment was pre-rinsed with Milli-Q water [71]. Samples and filters were kept covered with aluminum foil whenever possible beforehand, and work surfaces were cleaned before processing began [71,72]. Non-synthetic lab coats were worn. However, since quantitative field and procedural gaps were not made, we could not completely prevent airborne contamination (especially fibers) if fresh samples were taken.

### 2.4. SEM–EDX Sample Processing and Analysis

In the analyses performed using the Zeiss Sigma-300 field-emission scanning electron microscope (FESEM) (Carl Zeiss Microscopy GmbH, Oberkochen, Germany) and energy-dispersive X-ray spectrometer (EDX), only microplastic (MP) samples larger than 300 µm were evaluated due to technical difficulties in using the device. Each collected sample was first examined under a stereo microscope and selected using fine-tipped tweezers, and then placed on a 1 × 1 cm carbon tape attached to SEM metal pins. The samples were coated with a thin layer of gold using a Quorum SC7620 Sputter Coater (Quorum Technologies Ltd., Laughton, UK) at a pressure of 8 millibars. Subsequently, the MP particles were imaged on the TESCAN Vega 3 SEM at an accelerating voltage of 5.0 kV and magnification ratios ranging from 85× to 2840× [42]. For EDX analysis, two different points were marked on each sample, and the corresponding EDX spectra were obtained.

### 2.5. Fourier Transform Infrared Spectrophotometer (FTIR) Analysis

For FTIR analyses, very small particles may lead to weak absorbance signals. Sizes of 300 µm and above allow sufficient characteristic data (signals) to reach the instrument’s detector, helping to create clearer and more distinctive peaks. It has also been observed that very small particles are quite difficult to remove from the filter papers, and losses occur during the collection process. To identify polymer types using Fourier Transform Infrared (FTIR) Spectrophotometry [73], only selected MP fibers longer than 300 µm were used due to the difficulties in processing numerous MPs and smaller MPs. The chemical bonds in the particles were identified using a Bruker ALPHA Fourier Transform Infrared Spectrophotometer (FTIR). Approximately 20% of the total particles were subjected to FTIR analysis. The optical properties of the pigments (UV absorption) were measured using a spectrometer.

### 2.6. Statistical Analysis

The relationship between regions and microplastic characteristics was examined through cross-tabulations and statistically evaluated using the Pearson chi-square test. This test assessed the significance of the difference between observed and expected frequencies to determine whether the distribution was driven by random chance or systematic environmental factors. All statistical associations were evaluated at a significance level of *p* < 0.05.

## 3. Results

### 3.1. Abundances and Distributions of MPs

Our results show that the feces of Armenian gulls collected from 4 stations in the Van Lake Basin were contaminated by MPs (size < 5000 µm) (see Table 1). From 480 fecal deposits (pooled into four composite sets), a total of 8197 MP items were identified. These data equate to a mean abundance of 17.07 items per deposit, with values ranging between 9.87 and 29.5 based on composite-derived estimates.

### 3.2. MP Particle Size

**Figure 2:**
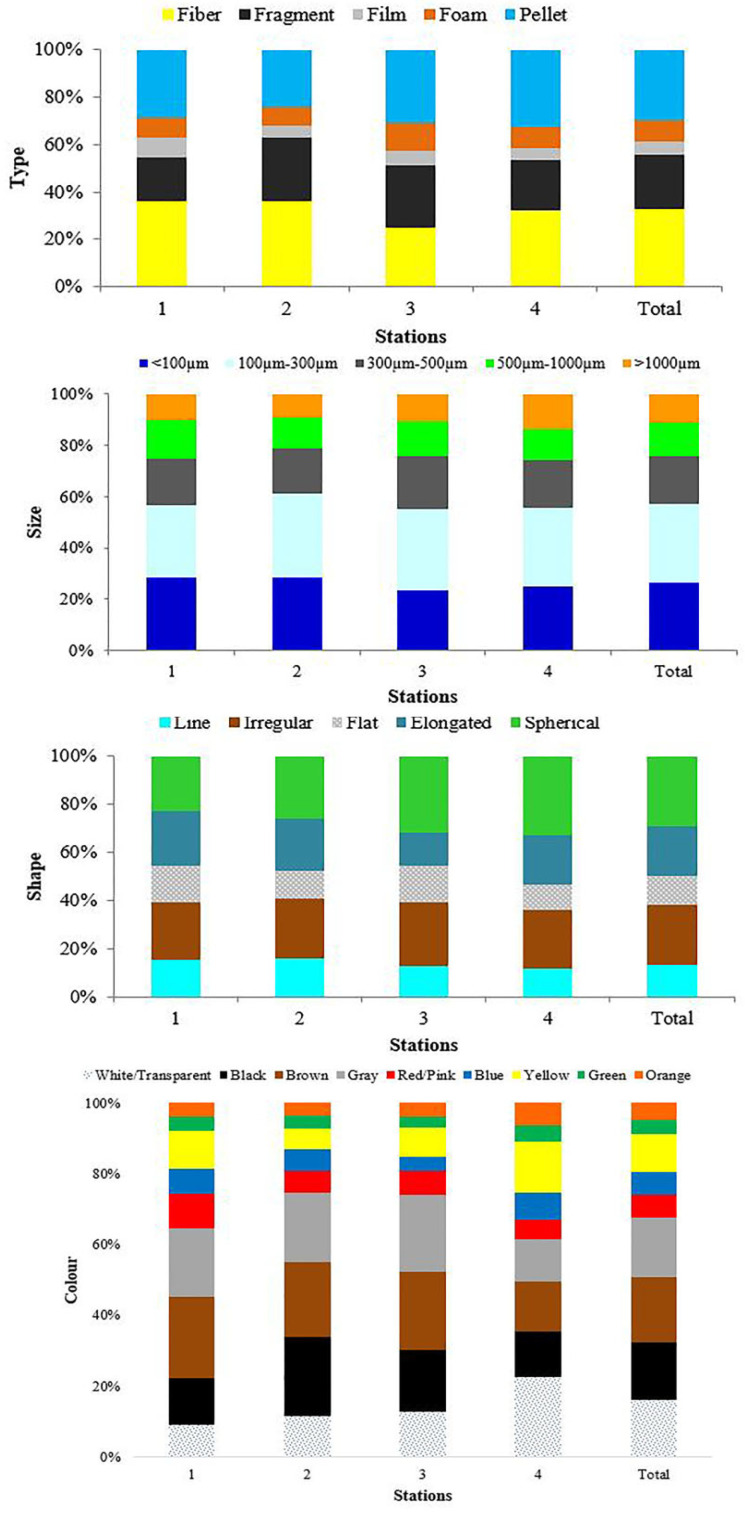
Distributions of type, particle size, shape and color of microplastics in Armenian gull feces.

In all fecal samples, MP particles within the 100–300 µm size class (2.524 MP) were identified as the most dominant category. This was followed by MP particles in the <100 µm (2.152 MP), 300–500 µm (1.538 MP), and 500–1000 µm (1.052 MP) size classes. Finally, MPs in the >1000 µm (931 MP) size class represented the smallest portion of the distribution (see Figure 2, Table 2). The findings indicate that microplastic shapes significantly differ across regions (χ^2^ = 125,057; sd = 12; *p* < 0.001) (Table 3, Figure S1). Spherical and irregular shapes constitute the most common morphological groups across all regions. High proportions of spherical (32.6%) and irregular (24.7%) microplastics were observed in the Sıhke sampling site, while the Adalar region exhibited a higher rate of spherical microplastics (31.6%). It was determined that irregular shapes (23.8%) were more dominant in the Kampus region, whereas spherical shapes (25.9%) prevailed in the Kale region. These findings reveal that the distribution of microplastic shapes varies depending on regional characteristics (Table S1).

### 3.3. MP Type

**Figure 3:**
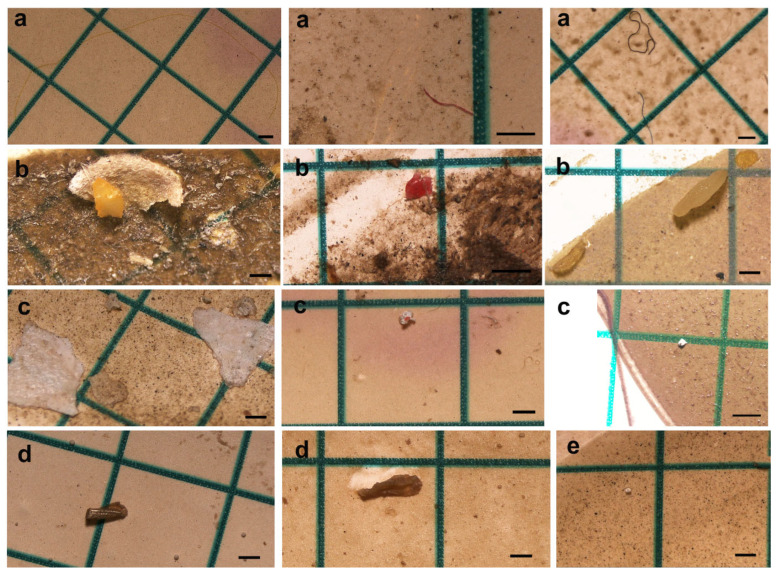
Photographs of fiber (**a**), fragment (**b**), foam (**c**), film (**d**), and pellet (**e**) microplastics observed in Armenian gull fecal samples (scale bar = 500 µm).

**Figure 4:**
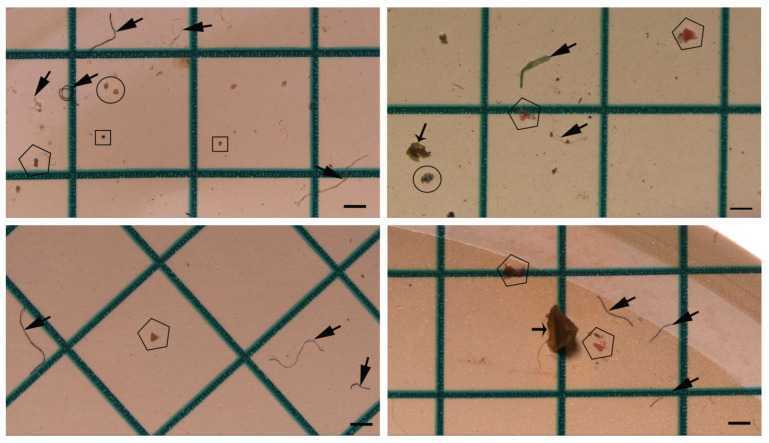
Photographs of fiber (Thick arrow), fragment (Pentagon), foam (Circular), film (Thin arrow), and pellet (Square) microplastics observed in Armenian gull fecal samples (scale bar = 500 µm).

The collected plastic particles were classified into distinct morphological categories, including fiber (Figure 3a, see Figure 4 (Thick arrow)), fragment (Figure 3b, see Figure 4 (Pentagon)), foam (Figure 3c, see Figure 4 (Circular)), film (Figure 3d, see Figure 4 (Thin arrow)) and pellet (Figure 3e, see Figure 4 (Square)). Fiber was identified as the most dominant microplastic (MP) type in the fecal samples, with a total count of 2.672 particles. In contrast, film was determined to be the least prevalent type, consisting of 451 particles (Figure 4). Other MP types were recorded in the following order of abundance: pellets with 2.426 particles, fragments with 1.917 particles, and foams with 731 particles (see Figure 2, Table 3).

Findings indicate statistically significant variations in microplastic composition between regions (χ^2^ = 138,811; sd = 12; *p* < 0.001) (Table 2, Figure S2). While fibers and pellets emerged as the most prevalent types across all sites, their relative proportions exhibited regional fluctuations. Specifically, the Sıhke site was characterized by a dominance of fibers (31.9%) and pellets (32.6%). In contrast, the Adalar site showed higher concentrations of fragments (26.7%) and pellets (31.1%) compared to other locations. In the Campus and Kale regions, fibers remained the predominant microplastic form. These results suggest that the distribution of microplastic types is fundamentally influenced by site-specific regional characteristics (Table S2).

### 3.4. MP Shape

In our study on Armenian gull feces, MPs were classified into five different categories: line, irregular, flat, elongated, and spherical. Within these classes, spherical-shaped plastics (2396 MPs) were found to constitute the most dominant shape class, with an average frequency of 29.2% among the MP shapes examined. Looking at the other shapes, they were counted as irregular (2032), elongated (1667), line (1109), and flat (993) (see Figure 2, Table 3). The findings demonstrate that microplastic shapes significantly differ across regions (χ^2^ = 125,057; sd = 12; *p* < 0.001) (Table 2, Figure S3). Spherical and irregular shapes constitute the most common morphological groups in all regions. It was observed that spherical (32.6%) and irregular (24.7%) microplastics were present in high proportions in the Sıhke sampling site, while the Adalar region exhibited a higher rate of spherical microplastics (31.6%). Irregular shapes (23.8%) were found to be more dominant in the Kampus region, whereas spherical shapes (25.9%) prevailed in the Kale region. These findings reveal that the distribution of microplastic shapes varies depending on regional characteristics (Table S3).

### 3.5. MP Color

The microplastics (MPs) identified in the fecal samples exhibited a diverse color spectrum, including white or transparent, black, brown, gray, red or pink, blue, yellow, green, and orange. Brown (1511 MPs) was determined to be the most prevalent color, followed closely by gray (1370 MPs), black (1332 MPs), and white or transparent (1322 MPs) variants, which together constituted the majority of the detected particles. In contrast, other color categories were observed with lower frequencies, recorded in descending order as yellow (874 MPs), blue (539 MPs), red or pink (527 MPs), orange (399 MPs), and green (323 MPs) (see Figure 2, Table 3). The findings indicate that the color distribution of microplastics differs significantly across regions (χ^2^ = 587,879; sd = 24; *p* < 0.001) (Table 2, Figure S4). In general, white/transparent, black, brown, and gray microplastics constitute the most common color groups in all regions. The Sıhke region particularly stands out regarding white/transparent (22.6%) and yellow (14.5%) microplastics. In the Kampüs, Kale, and Adalar regions, black, brown, and gray colors were found to be more dominant. Such differences in color distribution may be associated with the sources of the microplastics or their processes of environmental exposure (Table S4).

### 3.6. Polymer and SEM Analysis

**Figure 5:**
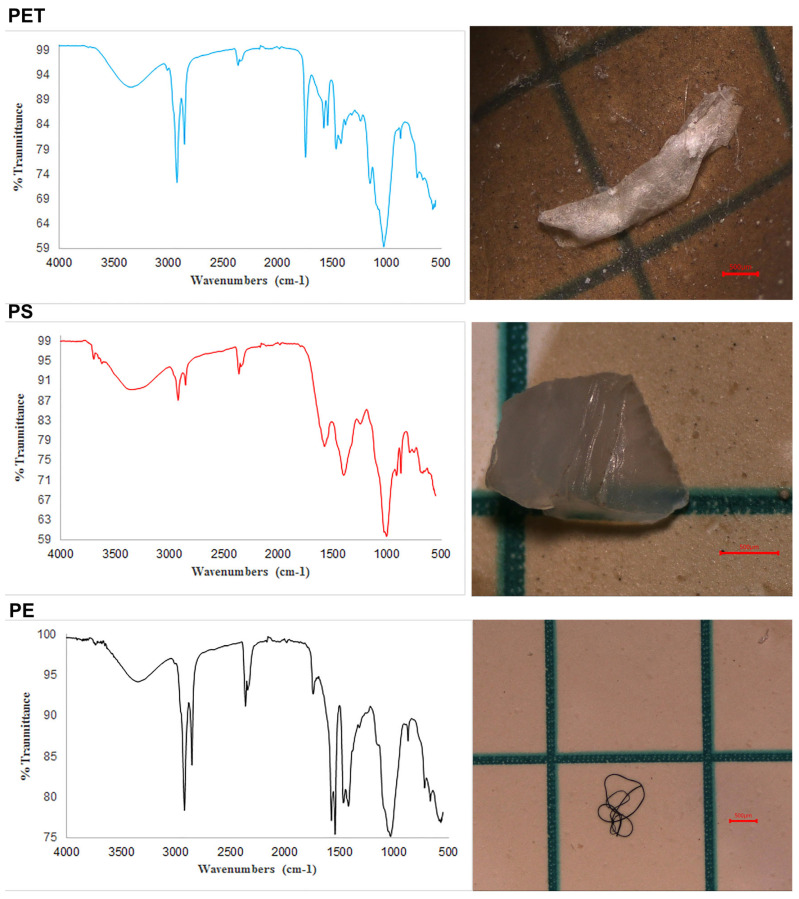
Characterization of MPs using FTIR—Polyethylene terephthalate (PET), polystyrene (PS), polyethylene (PE) (scale bar = 500 µm).

When examining the FTIR analysis results of Armenian gull fecal samples, three different polymer types were identified: polyethylene (PE) (42.6%), polystyrene (PS) (28.38%), and polyethylene terephthalate (PET) (8.5%). Other undetectable polymer types constitute 20.52% of the total. Images of MPs with chemical structures of polyethylene (PE), polystyrene (PS), and polyethylene terephthalate (PET) are shown in Figure 5. In our study, PE percentages were predominant in the samples. PE is most commonly used in plastic bags, agricultural materials, food packaging products, and plastic bottles, while PS is preferred in food packaging, plastic containers, pipes, and carpets. The Aldrich Organometallic, Inorganic Spectral Library was used to determine the chemical structures of the particles.

**Figure 6:**
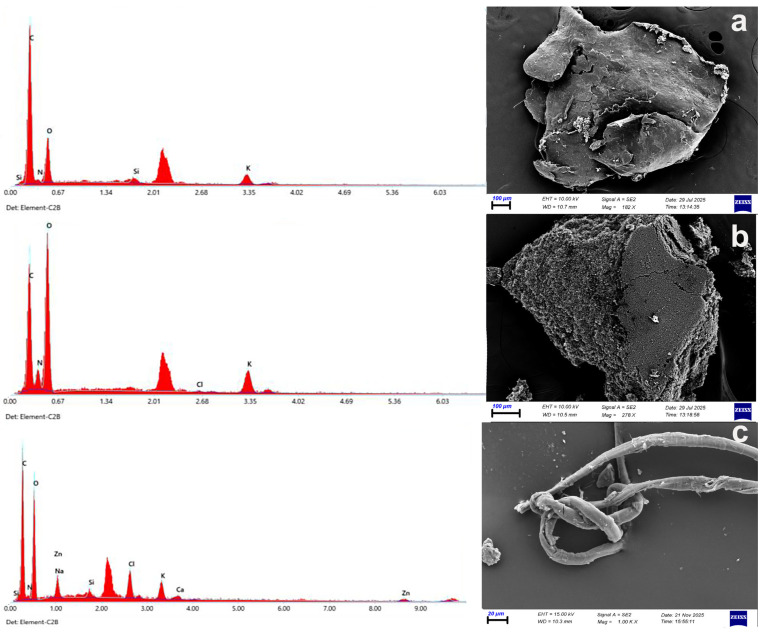
SEM images and elemental composition of the MPs: film (**a**), fragment (**b**) and fiber (**c**).

Looking at the SEM image, the polyethylene (PE) surface is relatively flat. However, physical wear and tear resulting from the removal of microplastics from feces is observed. Additionally, there are cavities and holes inside due to environmental effects. The main signal for PE is expected to be carbon (C) (see Figure 6c). Microcracks, fracture lines, and partially lost and worn surface flatness are present in the polyethylene terephthalate (PET) parts. Carbon (C) and oxygen (O) elements are the main signals for PET (see Figure 6a). The polystyrene (PS) particles in the SEM image exhibit a rough, porous, and irregular morphology. The observed fractured structures indicate that PS undergoes chemical and mechanical degradation due to environmental effects and the digestive system. Clustered dense areas indicate biofilm formation, and microorganisms are also seen to attach to the polystyrene surface (see Figure 6b).

## 4. Discussion

Evidence from inland and terrestrial avifauna shows that microplastics and microscopic anthropogenic debris can be detected using non-invasive or diet-linked matrices across different trophic guilds relevant to the Lake Van Basin. For example, ingestion of microscopic anthropogenic waste has been documented in small terrestrial passerines in Europe [71], and microplastics have been quantified in feces of European shags from Norway [72]. Complementary raptor-based evidence further indicates that pellet matrices can trace prey-mediated contamination and that extraction/processing choices can influence recovery, supporting careful method harmonization [73,74]. Closer to our study system, microplastics were reported in Greylag Goose feces from Lake Erçek [75], while wetland bird communities in Gharana Wetland and migratory shorebirds along the Central Asian Flyway also showed substantial occurrence and characteristic particle profiles [76,77].

Here, our study extends fecal-based MP monitoring to the Armenian gull (*Larus armenicus*) breeding in the Lake Van Basin, providing a baseline for an endorheic lake system where plastic inputs, circulation, and retention dynamics may differ from those of open coastal systems. Comparable exposure patterns have been documented elsewhere; for example, Matos et al. (2024) [74] reported similar MP exposure rates (30–49%) in both juvenile and adult Cape Verde shearwaters and Bulwer’s petrels. In addition, Le Guen et al. (2020) [75] found that 77% of fibers detected in king penguin feces from South Georgia were microfibers, with most reported as natural in origin—highlighting both the ubiquity of fiber contamination in remote ecosystems and the importance of polymer verification when interpreting “microfiber” results. Accordingly, interpretation of spatial differences in our dataset should be grounded in standardized QA/QC procedures and, where feasible, hypothesis-driven statistical comparisons among stations to robustly attribute variation to local pressures and waste inputs. According to the data obtained, a total of 8197 microplastic particles were detected in 480 fecal samples; an average of 9.87–29.5 particles per sample was found. This prevalence is very close to the findings of Heim et al. (2025) [39], who reported a 98% positivity rate in urban wetland birds, and Mendez-Sanhueza et al. (2023) [76], who reported a high frequency of MPs in seabird feces along the Chilean coast. Reetika et al. (2024) [77] identified MPs in fiber form in the majority of fecal samples (approximately 70%) in their study of different bird species in the Gharana Wetland, and determined high microplastic concentrations, particularly in the Bar-headed Goose (*Anser indicus*) and Purple Moorhen (*Porphyrio poliocephalus*) species during the winter season. These findings indicate that the high MP frequency observed in Lake Van samples (Atici et al. 2022) [58] may also be affected by seasonal and species factors. In the study conducted by Qiu et al. (2025) [78] on little egrets (*Egretta garzetta*) on Hainan Island, high MP concentrations were reported in rice field sediments and water, and it was determined that the feces were dominated by polymers (PE), polypropylene (PP), polyvinyl chloride (PVC), polyamide (PA), polyethylene terephthalate (PET) and polystyrene (PS), most of which were ≤100 μm in size and in fragment form. These results indicate that waterfowl living in agricultural areas may play a critical vector role in MP transport and that similar accumulation processes may occur in wetlands under anthropogenic pressure, such as Lake Van. Additionally, Jiang et al. (2024) [79] detected MPs in 37% of fecal samples collected from eight different bird species in China’s Yancheng Wetland area and reported the highest values in water birds; this indicates that habitat type is a determining factor in exposure. These results indicate that MPs are found at high frequency in bird feces and that fecal analysis is a reliable method to assess environmental exposure of birds. In our current study, it was determined that fiber (32.6%), pellet (29.6%) and spherical particles (29.2%) were dominant in Armenian gull fecal samples. This distribution is similar to that in the study by Gil-Delgado et al. (2017) [67], who identified fibers as the most common form in the feces of wetland birds in Spain, accounting for 68% of total particles. Similarly, Mendez-Sanhueza et al. (2023) [76] reported a fiber content of 83% in Chilean waters. Lourenço et al. (2017) [30] found microfibers in approximately 49% of fecal samples collected from three different African and European wetlands, noting that more than half of the fibers were synthetic polymers (particularly PET and polyacrylonitrile (PAN)). This study demonstrates that the dominance of the fiber form in different geographical regions is based on both anthropogenic and natural sources. In a study conducted by Bange et al. (2023) [80] on the Wadden Sea, fiber ratios were found to be between 74% and 76% for the common eider (*Somateria mollissima*) and common shelduck (*Tadorna tadorna*). These results are consistent with the fiber-weighted form distribution determined in our study. In line with this rationale, the Pearson chi-square analyses provide explicit evidence that MP characteristics are not homogeneously distributed among sampling stations. Specifically, both MP type composition (χ^2^ = 138,549; df = 12; *p* < 0.001) and shape categories (χ^2^ = 125,057; df = 12; *p* < 0.001) varied significantly across the four sites, indicating that the observed patterns are unlikely to reflect random variation alone. The station-level profiles suggest distinct source and processing signals: Sıhke was characterized by comparatively high proportions of fibers and pellets and a pronounced representation of spherical particles, whereas Adalar showed relatively higher contributions of fragments/pellets alongside elevated spherical particles; Campus displayed a greater prominence of irregular shapes, consistent with local fragmentation processes; and Kale remained dominated by fibers with a comparatively higher proportion of spherical particles. Together, these statistically supported differences are consistent with the expectation that site-specific land use, waste inputs, and local hydrodynamics in an endorheic basin can preserve “local signatures” of MP exposure, rather than yielding a uniform signal across the Lake Van system.

The dominance of the fiber form suggests that birds interact with freshwater environments and urban wastewater, particularly those enriched with textile fibers [81]. However, the high proportion of pellets detected along with fibers in our study suggests that plastic sources in the region are not limited to textile and household waste, but industrial granules and packaging materials also contribute. This finding is in line with the study by Perold et al. (2025) [82], who reported high proportions of industrial pellets in the feces of seabirds in the South Atlantic. Similarly, Veríssimo et al. (2025) [83] reported in their study conducted in the Ria Formosa region of Portugal that fiber and small plastic particles were predominant in Yellow-legged gulls (*Larus michahellis*) and little terns (*Sternula albifrons*), with blue and transparent pieces standing out in terms of color. The above results support the similar color and form distributions observed in our study. Fibers dominated the particles observed, which is consistent with many feces-based studies and may reflect widespread textile- and rope-derived inputs transported via wastewater, surface runoff or atmospheric deposition [20,39]. The relatively high proportion of pellet/spherical particles suggests that sources may also include industrial raw materials and packaging-related debris. However, without concurrent environmental sampling and source apportionment, these interpretations should be treated as plausible rather than definitive.

When the color distribution was examined, it was seen that brown, gray, black and transparent parts were dominant. This color profile is consistent with the study by Gil-Delgado et al. (2017) [67], who reported black and white particles as the most common colors in Spanish wetland birds. Additionally, Maaseide et al. (2024) [84] reported the dominance of blue and white MPs in the feces of European cormorants in Norway. Wei-Ting et al. (2025) [85] similarly reported in their study of different wetlands in Taiwan that polymers such as cellophane, polyester, and polyamide were found in high concentrations, with blue and white microplastics being the dominant colors.

Beyond the overall dominance of brown/gray/black/transparent particles, color composition also exhibited a clear station effect. The chi-square test indicated that MP color distributions differed significantly among regions (χ^2^ = 587,879; df = 24; *p* < 0.001), with Sıhke Lake standing out in particular for a higher representation of white/transparent and yellow particles, while Campus, Castle, and islands were comparatively enriched in darker tones (black, brown, gray). Such spatially structured color profiles may reflect differences in dominant source materials (e.g., packaging-related items versus more weathered particles), differential residence times and abrasion/soiling processes, and/or varying contributions from wastewater and runoff pathways. Therefore, the statistically supported color heterogeneity reinforces the interpretation that local inputs and environmental processing jointly shape the MP signal captured by fecal samples.

Color differences may vary depending on the areas of use of plastics; for example, blue fibers generally originate from textile products, while white and transparent particles originate from food packaging [77]. Plastic bags and packaging waste commonly used in settlements around Lake Van may explain the color distribution identified in our study. In terms of size distribution, the densest range was 100–300 µm (30.8%), and the <100 µm class was represented by 26.3%. This finding is similar to that of Maaseide et al. (2024) [84] in Norway, who reported that most of the particles were below 300 µm. Similarly, in a study conducted by Zhou et al. (2025) [86] in South China, particles between 100 and 500 µm constituted the majority of fecal samples. This dominance of small-sized particles can be attributed to both the abundance of degraded MPs in the environment and the fact that birds can easily ingest smaller particles through their diet. However, since FT-IR analysis could only be applied to particles ≥300 µm in our study, smaller particles could not be chemically verified. This situation may lead to an underestimation of the environmental PM load, as noted by Wang et al. (2025) [87].

In terms of polymer composition, our study found polyethylene (PE) at 42.6%, polystyrene (PS) at 28.4%, and polyethylene terephthalate (PET) at 8.5%. This distribution is largely parallel to studies conducted in different regions. For example, Athira et al. (2025) [81] identified PE as the dominant polymer in Indian shorebirds, while [82] Perold et al. (2025) reported that PE and PP were the most prevalent polymers in seabird feces from the South Atlantic. The high PS ratio indicates the character of local plastic resources [66]. Atici et al. (2021; 2022) [58,88] stated in their studies in Türkiye that PS particles originating from food packaging and foam materials are an important source of pollution in coastal areas. In this context, the dominant use of packaging and disposable products around Lake Van can be considered one of the reasons for the high PS rate in our study.

In our study, the high detection rate achieved with fecal samples supports the reliability of this method. Furthermore, a study conducted by Deoniziak et al. (2022) [37] on thrush species demonstrated that the dominance of fibers indicates that MPs are not solely of marine origin but can also be dispersed through atmospheric transport and terrestrial sources. This situation indicates that MP accumulation in semi-enclosed basins such as Lake Van is fed by both aquatic and terrestrial inputs. Our current findings can also be related to experimental findings in the literature regarding the potential effects of MPs on bird physiology. De Souza et al. (2022) [44] showed that aged polystyrene MPs caused oxidative stress and decreased antioxidant enzyme activities in Japanese quails. Similarly, Liu et al. (2025) [89] reported that MP intake causes disturbances in energy metabolism and gut microbiota. These findings suggest that the high PS rate detected in our study is not only an environmental signal but also important in terms of potential physiological effects. Studies conducted in different ecosystems in the literature reveal that differences in polymer composition and form distribution are mostly due to regional plastic use, settlement density, and waste management practices [76,77]. Around Lake Van, anthropogenic sources such as agricultural plastic film, single-use products, and textile waste can be counted among the main causes of this diversity. In addition, water birds’ position at the top of the food chain plays an important role in the transport of MPs through food and their release back into the environment via feces [39,40].

The pronounced station-level differences in MP type and shape composition observed across the Lake Van Basin indicate that MP exposure in Armenian gulls is shaped by localized waste inputs and retention/processing dynamics in an endorheic system. From a seabird protection perspective, this supports routine supervision of breeding and roosting hotspots, including periodic shoreline litter audits and fecal-based MP monitoring as an early-warning indicator. To enable robust trend detection and to evaluate mitigation effectiveness over time, monitoring should be standardized across seasons and stations (sampling design, contamination controls, particle classification, polymer verification, and reporting), using fecal-based approaches that are increasingly adopted as a non-invasive monitoring tool [38] and aligning interpretation with a One Health perspective on microplastics [19]. For the Lake Van Basin specifically, coupling avian monitoring with existing evidence of MP contamination in local surface waters can provide a coherent, basin-scale baseline for targeted management actions [57].

Reducing human-generated waste at source is therefore central to mitigating seabird exposure in the basin. Given the proximity of key sampling areas to recreation/tourism zones and solid-waste handling infrastructure, practical priorities include: (i) strengthening municipal solid-waste collection and containment (preventing open dumping and wind dispersal from transfer/landfill areas), (ii) enforcing “leave-no-trace” practices and installing adequate waste infrastructure at shoreline recreation sites and breeding islands, (iii) improving capture of lightweight plastics and microfibers via wastewater controls where feasible, and (iv) implementing source-reduction policies that curb single-use plastics and promote circular-economy solutions. These interventions are consistent with UNEP assessments emphasizing upstream leakage prevention and systemic waste management improvements as the most effective pathway to reduce plastic pollution loads in aquatic ecosystems [9,10].

## 5. Limitations of the Study and Recommendations

Although our results provide evidence of MP occurrence in *L. armenicus* feces, several limitations should be considered. First, polymer verification by FTIR/SEM–EDX was restricted to particles ≥300 µm, so the composition of smaller particles could not be confirmed and total MP loads may be underestimated [73,84]. Second, a portion of particles was classified visually, which can lead to misidentification of natural fibers or non-plastic particles without spectroscopic confirmation [70]. Third, to manage sample volume, fecal material was pooled into 16 composites, limiting interpretation at the individual level.

Because microplastics are ubiquitous in laboratory air and reagents, rigorous QA/QC (e.g., field/procedural blanks, recovery tests and contamination controls) is essential for comparability and data reliability [20,72,90]. We recommend explicitly reporting blank outcomes and applying blank corrections where appropriate. In addition, some particles could not be chemically identified and may include organic fibers or other anthropogenic microparticles [86]. Finally, sampling covered only specific periods, so seasonal variability associated with diet and migration could not be resolved [43].

Future work should (i) extend polymer identification to smaller size fractions using µ-FTIR/Raman, (ii) incorporate standardized QA/QC and recovery tests, (iii) increase temporal replication, and (iv) apply hypothesis-driven statistical comparisons among stations (to be added) to relate MP loads to environmental pressure gradients and bird ecology.

## 6. Conclusions

The high abundance of MPs identified in Armenian gull feces, the morphology of the fiber + pellet mixture, and the dominance of PE/PS exhibit a pattern consistent with feces-based bird studies worldwide. However, the relatively high PS and pellet ratios reflect regional plastic consumption and waste management practices. Fecal-based monitoring is a robust, non-invasive method for monitoring MP exposure in bird populations; however, future efforts should focus on detecting small-sized particles and determining toxicological effects.

## Figures and Tables

**Table 1:** Data from Armenian gull fecal samples.

Stations No.	Stations	Number of Samples	Number of Randomly Selected Samples	Number of Homogenized Samples	Total (MPs)
1	Kampüs	128	120	4	1185
2	Kale	198	120	4	2238
3	Adalar	132	120	4	1234
4	Sıhke	265	120	4	3540
	All Stations (Total)	723	480	16	8197

**Table 2:** Characteristics and distribution of MPs from Armenian gull feces.

Characteristics	Total/Mean Microplastic Density				
Size	<100 µm	100 µm–300 µm	300 µm–500 µm	500 µm–1000 µm	>1000 µm
	2152 ± 63.29	2524 ± 73,24	1538 ± 45.23	1052 ± 30.94	931 ± 27.38
	26.25%	30.79%	18.77%	12.84%	11.35%
Type	Fiber	Fragment	Film	Foam	Pellet
	2672 ± 78.55	1917 ± 56.38	451 ± 13.26	731 ± 21.52	2426 ± 71.41
	32.60%	23.38%	5.50%	8.92%	29.60%
Shape	Line	Irregular	Flat	Elongated	Spherical
	1109 ± 32.61	2032 ± 59.76	993 ± 29.2	1667 ± 49.02	2396 ± 70.47
	13.53%	24.78%	12.12%	20.34%	29.23%
Color	White/Transparent	Black	Brown	Gray	Red/Pink
	1322 ± 38.88	1332 ± 39.17	1511 ± 44.44	1370 ± 40.29	527 ± 15.5
	16.12%	16.24%	18.43%	16.72%	6.43%
	Blue	Yellow	Green	Orange	
	539 ± 15.85	874 ± 25.7	323 ± 9.5	399 ± 11.73	
	6.57%	10.67%	3.95%	4.87%	

**Table 3:** Results of chi-square (**χ**2) test between microplastic characteristics and sampling regions.

Variable (Feature)	Test Type	χ2 Value	df	*p*-Value	N
Type × Region	Pearson chi-square	138.811	12	<0.001	8.197
Shape × Region	Pearson chi-square	125.057	12	<0.001	8.197
Size × Region	Pearson chi-square	59.226	12	<0.001	8.197
Color × Region	Pearson chi-square	587.879	24	<0.001	8.197

## Data Availability

The original contributions presented in this study are included in the article/Supplementary Materials. Further inquiries can be directed to the corresponding author.

## Supplementary Materials

The following supporting information can be downloaded at https://www.mdpi.com/article/10.3390/toxics14030202/s1. Figure S1: Statistical comparison (chi-square tests) of the distribution of MP sizes across stations. Table S1: Distribution of MP sizes in sampling areas. Figure S2: Statistical comparison (chi-square tests) of the distribution of MP types across stations. Table S2: Distribution of MP types in sampling areas. Figure S3: Statistical comparison (chi-square tests) of the distribution of MP shapes across stations. Table S3: Distribution of MP shapes in sampling areas. Figure S4: Statistical comparison (chi-square tests) of the distribution of MP colors across stations. Table S4: Distribution of MP colors in sampling areas.
