# Supplementary material for: First Report on Occurrence and Characterization of Microplastics in Feces of Larus armenicus (Buturlin, 1934) in the Lake Van Basin (Eastern Anatolia, Türkiye)
Source: Toxics. 2026 Feb 27;14(3):202. doi: 10.3390/toxics14030202 (PMC13029877; doi:10.3390/toxics14030202)
Supplement: Supplementary file 1 [file toxics-14-00202-s001.zip › toxics-4160525-supplementary.pdf]

Chi-Square Tests

	Value	df	Asymp. Sig. (2-sided)
Pearson Chi-Square	59,226[a]	12	,000
Likelihood Ratio	58,839	12	,000
Linear-by-Linear Association	16,465	1	,000
N of Valid Cases	8197		

a. 0 cells (0,0%) have expected count less than 5. The minimum expected count is 134,59.

Figure S1. Statistical comparison (Chi-Square Tests) of the distribution of MP size across stations.

Table S1. Distribution of MP sizes in sampling areas

Size (µm)	Kampus n (%)	Kale n (%)	Adalar n (%)	Sıhke n (%)	Total n (%)
<100	338 (28.5)	637 (28.5)	291 (23.6)	886 (25.0)	2152 (26.3)
100–300	331 (27.9)	727 (32.5)	387 (31.4)	1079 (30.5)	2524 (30.8)
300–500	217 (18.3)	401 (17.9)	259 (21.0)	661 (18.7)	1538 (18.8)
500–1000	181 (15.3)	271 (12.1)	168 (13.6)	432 (12.2)	1052 (12.8)
>1000	118 (10.0)	202 (9.0)	129 (10.5)	482 (13.6)	931 (11.4)
Total	1185 (100)	2238 (100)	1234 (100)	3540 (100)	8197 (100)

Chi-Square Tests

	Value	df	Asymp. Sig. (2-sided)
Pearson Chi-Square	138,811[a]	12	,000
Likelihood Ratio	139,550	12	,000
Linear-by-Linear Association	30,271	1	,000
N of Valid Cases	8197		

a. 0 cells (0,0%) have expected count less than 5. The minimum expected count is 65,20.

Figure S2. Statistical comparison (Chi-Square Tests) of the distribution of microplastic type across stations.

Table S2. Distribution of Microplastic types in sampling areas

Type	Kampus Total MP (%)	Kale Total MP (%)	Adalar Total MP (%)	Sıhke Total MP (%)	Total n (%)
Fiber	431 (36.4)	805 (36.0)	305 (24.7)	1131 (31.9)	2672 (32.6)
Fragment	218 (18.4)	607 (27.1)	329 (26.7)	763 (21.5)	1917 (23.4)
Film	96 (8.1)	105 (4.7)	76 (6.2)	174 (4.9)	451 (5.5)
Foam	98 (8.3)	175 (7.9)	140 (11.3)	318 (9.0)	731 (8.9)
Pellet	342 (28.9)	545 (24.4)	384 (31.1)	1155 (32.6)	2426 (29.6)
Total	1185 (100)	2238 (100)	1234 (100)	3541 (100)	8197 (100)

Chi-Square Tests

	Value	df	Asymp. Sig. (2-sided)
Pearson Chi-Square	125,057[a]	12	,000
Likelihood Ratio	127,922	12	,000
Linear-by-Linear Association	41,167	1	,000
N of Valid Cases	8197		

a. 0 cells (0,0%) have expected count less than 5. The minimum expected count is 143,55.

Figure S3. Statistical comparison (Chi-Square Tests) of the distribution of MP shape across stations.

Table S3. Distribution of MP shapes in sampling areas

Shape	Kampus n (%)	Kale n (%)	Adalar n (%)	Sıhke n (%)	Total n (%)
Line	184 (15.5)	360 (16.1)	155 (12.6)	410 (11.6)	1109 (13.5)
Irregular	282 (23.8)	547 (24.4)	330 (26.7)	873 (24.7)	2032 (24.8)
Flat	179 (15.1)	268 (12.0)	185 (15.0)	361 (10.2)	993 (12.1)
Elongated	268 (22.6)	483 (21.6)	174 (14.1)	742 (21.0)	1667 (20.3)
Spherical	272 (23.0)	580 (25.9)	390 (31.6)	1154 (32.6)	2396 (29.2)
Total	1185 (100)	2238 (100)	1234 (100)	3540 (100)	8197 (100)

Chi-Square Tests

	Value	df	Asymp. Sig. (2-sided)
Pearson Chi-Square	587,879[a]	24	,000
Likelihood Ratio	593,333	24	,000
Linear-by-Linear Association	4,798	1	,028
N of Valid Cases	8197		

a. 0 cells (0,0%) have expected count less than 5. The minimum expected count is 46,69.

Figure S4. Statistical comparison (Chi-Square Tests) of the distribution of MP colour across stations.

Table S4. Distribution of MP colours in sampling areas

Colour	Kampus n (%)	Kale n (%)	Adalar n (%)	Sıhke n (%)	Total n (%)
White/Transparent	108 (9.1)	256 (11.4)	159 (12.9)	799 (22.6)	1322 (16.1)
Black	157 (13.2)	505 (22.6)	213 (17.3)	457 (12.9)	1332 (16.2)
Brown	270 (22.8)	472 (21.1)	274 (22.2)	495 (14.0)	1511 (18.4)
Gray	230 (19.4)	440 (19.7)	269 (21.8)	431 (12.2)	1370 (16.7)
Red/Pink	115 (9.7)	138 (6.2)	83 (6.7)	191 (5.4)	527 (6.4)
Blue	86 (7.3)	135 (6.0)	47 (3.8)	271 (7.7)	539 (6.6)
Yellow	124 (10.5)	132 (5.9)	105 (8.5)	513 (14.5)	874 (10.7)
Green	50 (4.2)	82 (3.7)	35 (2.8)	156 (4.4)	323 (3.9)
Orange	45 (3.8)	78 (3.5)	49 (4.0)	227 (6.4)	399 (4.9)
Total	1185 (100)	2238 (100)	1234 (100)	3540 (100)	8197 (100)
